# Effects of Different Extraction Methods in Pharmacopoeia on the Content and Structure Transformation of Ginsenosides

**DOI:** 10.3390/molecules27144347

**Published:** 2022-07-06

**Authors:** Hui Li, Hua Jiang, Lei Xu, Yaling Deng, Jing Xu, Yuqing Zhao

**Affiliations:** 1 School of Functional Food and Wine, Shenyang Pharmaceutical University, Shenyang 110016, China; 18844737860@163.com (H.L.); hahajiang6388@163.com (H.J.); 18246691730@163.com (L.X.); yl18341473234@163.com (Y.D.); 2 Key Laboratory of Natural Medicines of the Changbai Mountain, Ministry of Education, Yanbian University, Yanji 133002, China

**Keywords:** pharmacopoeia, extraction method, UPLC-Q-Exactive-MS, ginsenoside, response surface methodology

## Abstract

Ginseng (*Panax ginseng* C. A. Meyer), a perennial herb, possesses immunostimulatory, anticarcinogenic, antiemetic, and antioxidative biological activities. In recent years, more and more people have paid attention to the extraction methods and quality evaluation of ginseng. China, the United States, Europe, Japan, and Korea have all had the quality standards and content determination methods of ginseng. The different treatment methods are adopted before the determination of ginseng samples and the content limits of the index components, such as ginsenoside Rb_1_, ginsenoside Rg_1_, and ginsenoside Re exist differences. The similarities and differences of ginseng content detection methods in pharmacopoeias of different countries have been analyzed by a research group, but the comparison of the effects of different methods on the ginsenoside content and structural transformation has not been reported. In this paper, ginsenosides in ginseng were extracted according to four national pharmacopoeias and analyzed quantitatively and qualitatively by UPLC-Q-Exactive-MS and HPLC-UV. It was illustrated that the pretreatment method has a significant influence on the content determination of ginseng. The yield of rare saponins was increased by heating concluded from both the qualitative and quantitative comparison. Finally, a simple and feasible extraction method was optimized by response surface method at room temperature. The analysis of the preparation method and process optimization of the four pharmacopoeias can provide important reference information for the revision of ginseng standards.

## 1. Introduction

Ginseng (*Panax ginseng* C. A. Meyer) as a traditional herbal medicine occupies a prominent position in Chinese history. It is reported that the first country in the world to record ginseng is China [1]. A wide variety of ginseng products exist, with the health function of improving human immunity and combatting fatigue [2]. In the ginseng product industry, due to the uneven quality of ginseng, which results in different prices, it is imperative to carry out research on the quality of ginseng [3]. Ginsenosides are a family of triterpenoids with tetracyclic triterpenoid skeletons, which are widely regarded as the main active components of *Panax ginseng* [4,5]. There are two main dammarane-type triterpene saponins, including protopanaxatriol (PPT) and protopanaxadiol (PPD) [6]. Generally, ginsenosides Rb_1_, Re, Rd, Rc, Rg_1_, and Rb_2_ are the six major ginsenosides in ginseng [7]. Ginsenosides have neuroprotective effects and play a role in enhancing cognitive ability and memory [8,9,10]. At present, more than 200 kinds of ginsenosides and non-saponins have been isolated and identified from *Panax ginseng* [11]. Ginsenosides can be converted into secondary saponins and aglycones [12], which are easily absorbed by the human body during processing and play a role in superior biological activity in inhibiting tumor cells [13], improving cognitive ability [14], protecting lung function [15], and so on.

Modern studies have shown that ginsenoside content is an important index to measure the inherent quality of ginseng varieties [16,17]. The data show that the standardization index of ginseng products is based on the ratio of total ginsenosides to Rb_1_/Rg_1_. Asian ginseng is characterized by a Rb_1_/Rg_1_ ratio of 1 to 3, and American ginseng is characterized by a ratio of Rb_1_/Rg_1_ of 10 or more [18]. The ginseng quality standards specified in the US Pharmacopoeia (USP), the European Pharmacopoeia (EP), and the Japanese/Korean Pharmacopoeia (J/KP) are ginsenoside Rg_1_ and ginsenoside Rb_1_. The Chinese Pharmacopoeia (CP) of 2015 stipulates that the contents of ginsenosides Rg_1_, Rb_1_, and Re can be used as a standard for measuring the quality of ginseng [19,20,21,22]. Therefore, based on the comprehensive pharmacopoeias of various countries, ginsenoside Rg_1_ and Rb_1_ are selected as indicators for detection.

The CP, the USP, and the EP all involve heating. According to the research report, the thermal transformation of ginseng will bring about the change of content [23]. For example, white ginseng can be turned into red ginseng by heating, and the active components in red ginseng can be doubled by the transformation of white ginseng, especially the rare saponins [24,25,26]. The increase of ginsenoside content can enhance the activity of ginseng, but due to the deficiency of ginseng quantification method, the quality identification of ginseng is defective and cannot show the true quality of ginseng.

There have been research studies that analyzed the similarities and differences of ginseng content detection methods in pharmacopoeias of different countries, but the comparison of the effects of different methods on ginsenoside content and structural transformation have not been reported. In this paper, ginsenosides in ginseng were extracted according to CP, J/KP, USP, and EP, and analyzed quantitatively and qualitatively by UPLC-Q-Exactive-MS and HPLC-UV to investigate the effects of different extraction methods on the content and transformation of ginsenosides. At the same time, we studied a simple and feasible extraction method by response surface method at room temperature. The analysis of the preparation method and process optimization of ginsenoside can provide an important reference for the revision of ginseng standards.

## 2. Results and Discussion

### 2.1. Optimization of UPLC-Q-Exactive-MS Conditions

In order to obtain more ginsenosides, it is necessary to optimize the separation and detection conditions of the analytical method. The standard solution of ginseng sample was directly injected into UPLC-Q-Exactive-MS. The UPLC conditions were optimized to avoid ion suppression and improve the detection sensitivity. We studied the effects of four different ratios of methanol‚—water, methanol: 0.1% formic acid water, acetonitrile: water, and acetonitrile: 0.1% formic acid water—on the separation of ginseng. However, the pressure of methanol in organic phase is higher than that of acetonitrile liquid chromatography system, so we choose acetonitrile as the organic phase in mobile phase. The ratio of acetonitrile: 0.1% formic acid water was the best, so we chose acetonitrile: 0.1 formic acid water as the mobile phase for gradient elution.

### 2.2. UPLC-Q-Exactive-MS Analysis

In total, 36 ginsenosides were identified by UPLC-Q-Exactive-MS. Among them, 36 ginsenosides were identified by USP, 34 ginsenosides by CP, 21 ginsenosides by EP, and at least 19 ginsenosides were identified for unheated J/KP. In the process of extracting ginseng samples, CP, EP, and USP all involve the heating process, while J/KP is not heated, so J/KP has the least identified ginsenosides, and only Rg_3_ is a rare ginsenoside. However, a variety of rare saponins were detected in the other three pharmacopoeias. Therefore, we speculated that the original ginsenoside would be transformed into a variety of rare ginsenosides during heating.

**Figure 1:**
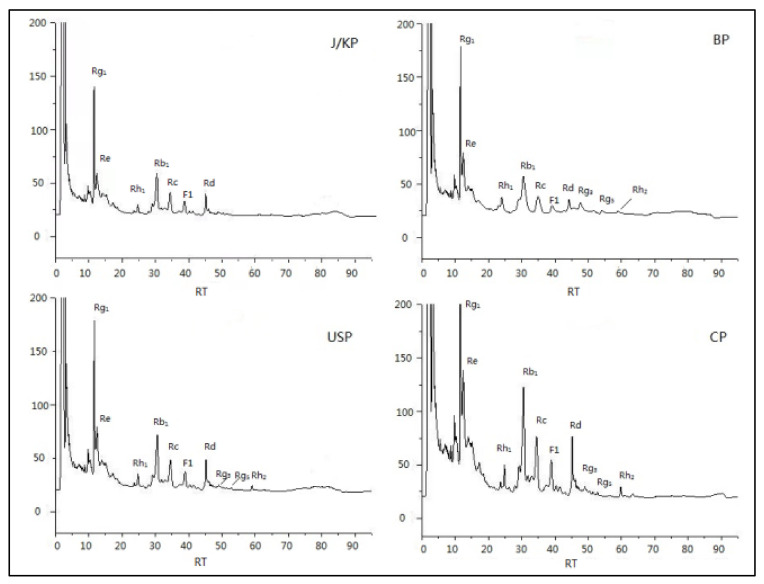
Quantitative chromatogram of HPLC analysis of ginseng using the four different countries’ pharmacopoeia extraction methods.

MS fragmentation and structure analysis [27]: the main structure of ginsenoside was PPD type and PPT type. PPD compounds are associated with the C-3 and/or C-20 positions. The glycosidic bond at the C-20 position is easily broken and a sugar molecule is lost. If it is a disaccharide, the two molecules are lost together. Secondly, the C-3 glycosyl group fell off in turn, accompanied by the loss of H_2_O molecules in the whole process. The PPT type is that the glycosyl group, which is linked to the C-6 and/or C-20 position. During the cleavage, the C-20 and C-6 sugars were lost first and then successively, accompanied by the loss of H_2_O molecules. The characteristic fragment information of PPD ginsenoside was M/Z 443, 425, and 407. Taking ginsenoside Rb_2_ as an example, its molecular weight is 1079.27, [M+Na]^+^, M/Z was 1101.5801, and its molecular formula is C_53_H_90_O_22_. Then, the secondary mass spectrum information was analyzed and was M/Z 767.4959 [M-glc-xyl+H]^+^, 749.4847 [mM-glc-xyl-H_2_O+H]^+^, 605.4420 [M-2glc-xyl+H]^+^, 587.4309 [M-2glc-xyl-H_2_O+H]^+^, 443.3875 [M-3glc-xyl+H]^+^, 425.3782 [M-3glc-xyl-H_2_O+H]^+^, and 407.3674 [M-3glc-xyl-2H_2_O+H]^+^, which accorded with the structural characteristics of ginsenoside Rb_2_. The characteristic fragment information of PPT ginsenoside is M/Z 441, 423, and 405. Taking ginsenoside Rh_1_ as an example, its molecular weight is 638.87, M/Z is 661.4286 [M+Na]^+^, and its molecular formula is C_36_H_62_O_9_. Then, the secondary mass spectrum information was analyzed and was M/Z 621.4354 [M-H_2_O+H]^+^, 603.4250 [M-2H_2_O+H]^+^, 459.3816 [M-glc+H]^+^, 441.3721 [M-glc-H_2_O+H]^+^, 423.3619 [M-glc-2H_2_O+H]^+^, and 405.3511 [M-glc-3H_2_O+H]^+^, which was consistent with the structural characteristics of ginsenoside Rh_1_. The identification results are shown in Figure 1, Figure S1 and Table S1.

Through analysis, it was found that the C-20 position deglycosylation reaction occurs first in the heated conversion process, and the deglycosylation products continue to be converted into other products, as shown in Figure S2. By summarizing the structural characteristics of the conversion products, the main reaction processes that occur in the heated conversion of dammarane saponins are obtained: (1) Deglycosylation reaction: preferential removal of the glycosyl substituent at the C-20 position; (2) dehydration reaction: C-20 hydroxyl group scavenging reaction with *β*-Hydrogen at C-21 or C-22 to generate isomers of Δ20(21) and Δ20(22); (3) cyclization reaction: C-20 hydroxyl group is added to C-24 and C-25 double bonds to form six membered rings containing an oxygen structure. In addition, some characteristic neutral losses were also observed, that is, the sapogenin ions C-24 and C-25 cleavage to produce 58 Da and the sapogenin ions C-20 and C-22 to produce 84 Da, which respectively represent the hydration reaction and structure of the olefin chain of the sapogenin. It can be inferred that the dammarane-type saponins underwent a cyclization reaction. The above results indicate that dammarane-type saponins can be effectively converted into rare saponins by heating, and HPLC-ESI-MS/MS is an effective method for analyzing the structure and pathway of saponins conversion products.

### 2.3. Determination of Ginsenoside Content

The contents of Ginsenoside Rg_1_, Re, Rh_1_, Rb_1_, Rc, F_1_, RD, Rg_3_, Rg_5_, Rh_2_, PPT, and PPD were calculated according to the external standard one point method. The liquid phase diagram is shown in Figure S3. Among the four extraction methods, Re, Rg_1_, and Rb_1_ were the most abundant. Among them, J/KP’s extraction method extracted the most original saponins, Re 2.25 mg/kg, Rg_1_ 0.70 mg/kg, and Rb 12.42 mg/kg. The rare saponins Rh_1_, F_1_, Rg_3_, Rg_5_, and Rh_2_ were extracted from the other three extraction methods, and the content of CP was the highest, at Rh_1_ 0.21 mg/kg, F_1_ 0.05 mg/kg, F1 0.05 mg/kg, Rg_3_ 0.02 mg/kg, Rg_5_ 0.09 mg/kg, and Rh_2_ 0.01 mg/kg, respectively. The specific content is shown in the Table 1.

### 2.4. Extraction Parameters

**Figure 2:**
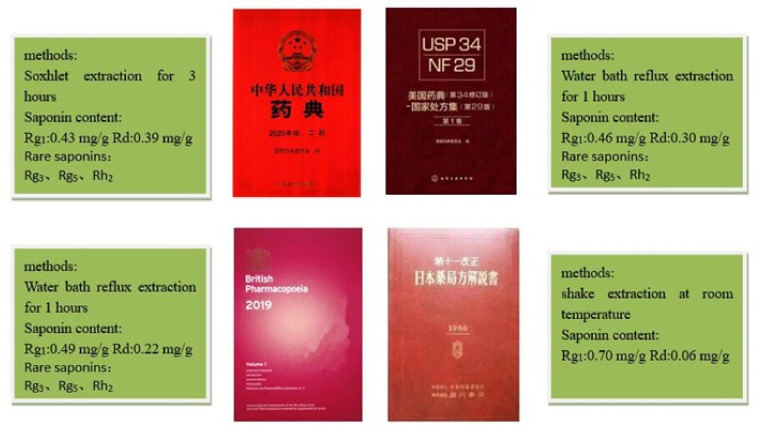
The different pretreatment methods in China Pharmacopoeia, Japan/Korea Pharmacopoeia, the United States Pharmacopoeia, and European Pharmacopoeia on ginsenosides.

The content of ginsenosides extracted by the ultrasonic method was affected by the type of solvent, the ratio of liquid to material, and the extraction time. In our study, the effects of solvent type, solvent concentration, liquid–solid ratio, and extraction time on the extraction rate of ginseng total saponins were investigated. As shown in Figure 2, all parameters significantly affected the extraction rate of ginsenosides in ginseng. Among the factors of solvent type, ethanol was the best solvent, and the content of ginsenoside was 28.45 mg/g. When 50–70% ethanol was used as solvent, the content of ginsenoside increased. The extraction time reached the peak in 30 min, and the total ginsenoside content was 25.16 mg/g. When the ratio of liquid to material is 20 mL/g and 30 mL/g, the total saponin content is 33.53 mg/g and 33.50 mg/g, and the extraction efficiency is higher. Based on the analysis of economy and other factors, the best level of this factor is the ratio of material to liquid of 20 mL/g.

### 2.5. Effects of the Extraction Variables on Total Ginsenosides Content

Taking ethanol concentration (A), liquid to material ratio (B), and extraction time (C) as independent variables, the determination result of total ginsenoside content in the test solution was used as the response value (Y), with the help of Design Expert 8.0.6.1 software. The BBD method was designed accordingly. Three factors and three levels were investigated. The content of total ginsenosides was calculated. The experimental design scheme and the experimental determination of the total saponin content are shown in Table 2.

The experimental data were analyzed by Design Expert 8.0.6.1 software, and the experimental data were subjected to regression fitting to obtain the quadratic regression equation with the total saponin content as the response value (Y).

Y = 41.92 + 1.79A + 0.002375B − 0.12C + 0.35AB + 1.08AC − 0.90BC − 6.59A^2^ − 3.30B^2^ − 3.16C^2^

The size and positive and negative of each coefficient in the equation indicate the degree and direction of the interaction of each factor, and each factor on the Y value is the total saponin content of ginseng. It can be seen from the formula that the factors most influencing the total ginsenoside content of ginseng are the ethanol concentration (A), the interaction term of ethanol concentration (A), the extraction time (C), and the second term of extraction time (C) on ginseng total. The effect of saponin content is inferior to ethanol concentration (A). The experimental data were analyzed by Design Expert 8.0.6.1 software, and the results of the variance analysis of the experimental regression equation are shown in Table 3.

It can be seen from Table 3 that the *p*-values of the secondary terms of ethanol concentration (A), ethanol concentration (A), liquid–material ratio (B) quadratic term, and extraction time (C) are all less than 0.01, indicating its impact on the response value is very significant. The *p*-value of the interaction term between ethanol concentration (A) and extraction time (C) is less than 0.05, indicating that the effect on the response value is significant.

The experimental model *p*-value < 0.0001 is extremely significant; the lack of fit *p*-value is 0.0697, which is greater than 0.05 and not significant, indicating that the experimental error is small; these two data indicate that the model design is reasonable. The experimental value R^2^ = 98.68% is greater than 90%; this value can indicate that the experimental model has a good fitting degree, and the predicted value of the model is highly consistent with the experimental results. The experimental method has high credibility and accuracy. A good experimental design, the results Adj R^2^ value should be very close to R^2^; the experimental Adj R^2^ is 96.97% and the R^2^ value is 1.71%, indicating that the experimental results do not agree with the model, since the value is only 1.71%, which is very small; it also shows that the experimental model is successfully modeled, and this model can be used to analyze and predict the extraction content of ginseng total saponins in ginseng.

### 2.6. Investigating the Interaction of Factors

The data were processed using software to predict the effect of three factors (solvent concentration, liquid to material ratio, and extraction time) on the total ginsenoside content of ginseng.

**Figure 3:**
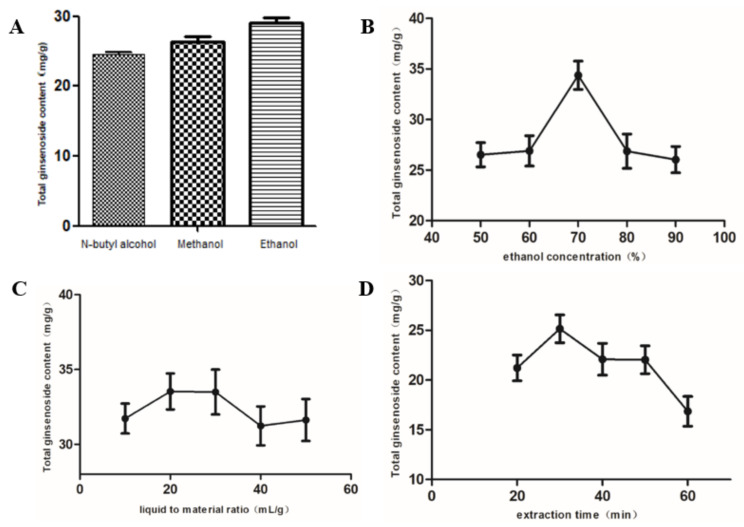
Effects of extraction solvent (**A**), extraction ethanol concentration (**B**), liquid–solid ratio (**C**), and extraction time (**D**). Data are presented as mean ± S.D. (*n* = 3).

It can be seen in Figure 3A that the interaction of ethanol concentration and liquid to material ratio affects the content of total ginsenosides in the extraction process. The concentration of ethanol is between 70% and 75%. When the ratio of material to liquid is between 20 and 25 mL/g, the extraction amount of total ginsenoside is the largest, and the ethanol concentration and liquid to material ratio are increased or decreased. The amount of extraction is gradually getting smaller. The ethanol concentration axis contour line is denser than the liquid material than the axis direction contour line, indicating that the ethanol concentration has a greater effect on the total ginseng content than the liquid to material ratio.

It can be seen in Figure 3B that the ethanol concentration and extraction time have a significant effect on the total saponin content of ginseng during extraction. The ethanol concentration was between 70% and 75%. When the extraction time was between 30 and 35 min, the total extraction amount of ginseng total saponin was the highest. The ethanol concentration and extraction time increased or decreased, and the extraction amount of total ginseng gradually changed. From the intensity of the contour line of the ethanol concentration axis and the contour line of the extraction time axis, the contour line in the ethanol concentration axis direction is denser, indicating that the effect of ethanol concentration on the total ginsenoside content is greater than the extraction time.

In Figure 3C, it can be seen that the ratio of liquid to material and extraction time has a significant effect on the total saponin content of ginseng during extraction. When the liquid to material ratio is between 20 and 25 mL/g and the extraction time is between 30 and 35 min and the total extraction amount of ginseng total saponins is the highest, regardless of the liquid to material ratio and extraction time and the extraction amount of total ginsenosides. Both are gradually decreasing. The contour map of the interaction between the two shows that the shape of the contour map is elliptical, indicating that the interaction between these two factors is significant [28].

### 2.7. Optimal Extraction Process Parameters and Experimental Verification of Response Surface

The 2D contour plots in Figure S4 showing the effects of ethanol concentration, liquid to material ratio, and extraction time on the extraction yield of ginsenoside content and their mutual effects.

The experimental optimal extraction process model conditions were ethanol concentration of 74.37%, liquid to material ratio of 21.07 mL/g, extraction time of 33.04 min, and theoretical optimal value of total ginsenoside content of 42.34 mg/g.

From the convenience of experimental operation and the accuracy of equipment and instrument, the optimal experimental conditions obtained by software simulation were adjusted to ethanol concentration of 74%, liquid to material ratio of 21 mL/g, and extraction time of 33 min. To confirm these results, five parallel analyses were evaluated at the optimal level. The average ginsenoside content of ginseng obtained was 42.34 mg/g, the SD value of the standard deviation of the experiment was 0.82%, and the relative standard deviation (RSD) of the experiment was 0.02%, indicating that the established model fits the actual condition better. The model is reliable.

In conclusion, HPLC-UV and UPLC-Q-Exactive-MS were used to analyze the treatment methods of ginseng in four pharmacopoeias in this study. In total, 36 ginsenosides were identified by UPLC-Q-Exactive-MS. Among them, 36 ginsenosides were identified by USP, 34 ginsenosides by CP, 21 ginsenosides by EP, and at least 19 ginsenosides were identified for unheated J/KP. In the process of extracting ginseng samples, CP, EP, and USP all involve the heating process, while J/KP is not heated, so J/KP has the least identified ginsenosides, and only Rg_3_ is a rare ginsenoside. However, a variety of rare saponins were detected in the other three pharmacopoeias. Therefore, we speculate that the original ginsenoside will be transformed into a variety of rare ginsenosides during heating. Twelve kinds of ginsenosides in ginseng were extracted using the methods of four national pharmacopoeias and quantitatively analyzed by HPLC-UV. The results showed that the content of rare ginsenoside was increased by heating, and the heating time was proportional to the content of rare ginsenoside. However, no rare saponins were detected in the unheated J/KP method. It is speculated that the original ginsenosides may be transformed into rare ginsenosides after heating. In the following study, UPLC-Q-executive-MS was used to analyze the pretreatment methods of ginseng in the four pharmacopoeias. The qualitative results of four pharmacopoeias showed that the content of ginsenoside inferred from CP, USP, and EP was higher than that of unheated J/KP. It was further verified that the original ginsenosides could be transformed into rare ginsenosides by heating.

The CP, EP, and USP all involve the heating process in the process of extracting ginseng samples. Heating can transform ginsenosides, thus affecting the accuracy of the experimental determination results. The J/KP extracts ginsenosides by shaking, and the extraction is incomplete. Based on the CP, in order to obtain the quality standard of ginseng with a high content of ginsenosides, one must avoid the change of saponin content caused by heating. Therefore, the single factor experiment method and the response surface method were used to optimize the extraction process of ginsenosides in ginseng at room temperature.

The regression analysis of the experimental results was done using Design Expert 8.6.0.1 software. The theoretical optimum conditions for extraction of ginsenosides were ethanol concentration of 74.37%, liquid to material ratio of 21.07 mL/g, extraction time of 33.04 min, and theoretical optimal ginsenoside content of 42.34 mg/g. Considering the convenience of experimental operation and the accuracy of equipment and instruments, the theoretical optimal conditions for ginseng saponin extraction in ginseng were adjusted. The ethanol concentration was adjusted from 74.37% to 74%, and the liquid to material ratio was adjusted from 21.07 mL/g to 21. In mL/g, the extraction time was adjusted from 33.04 min to 33 min. The experiment was carried out to verify that the method was reliable and could be applied to the actual situation. The actual extraction of total ginsenoside content was 42.34 mg/g.

## 3. Material and Methods

### 3.1. Reagents and Materials

A KQ3200DB ultrasonic cleaning bath (Kunshan Ultrasonic Instrument Co., Ltd., Shanghai, China) was used. Chromatographic grade methanol and acetonitrile were purchased from Fisher Scientific International Inc., (Hampton, NH, USA). Purified water was purchased from Wahaha (Hangzhou, China).

The ginseng sample (Huanren City, Liaoning Province) was identified as 15-year-old ginseng by Professor Lu Jincai of Shenyang Pharmaceutical University. A voucher specimen (PT 20200411) was kept in the herbarium of Shenyang Pharmaceutical University. The samples were dried at 45 °C to constant weight. Ginsenoside Rg_1_, Re, Rh_1_, Rb_1_, RC, F_1_, RD, Rg_3_, Rg_5_, Rh_2_, PPT, and PPD were self-made in the laboratory (purity > 98%).

### 3.2. Preparation of Standard Solutions and Sample Solutions

Ginseng was treated according to the methods of CP, USP, EP, and J/KP [19,20,21,22]. The main difference of the pre-processing is reflected in Table 4. We weighed 1 mg of 12 ginsenosides, Rg_1_, Re, Rh_1_, Rb_1_, Rc, F_1_, RD, Rg_3_, Rg_5_, Rh_2_, PPT, and PPD, to prepare 1 mg/mL solution.

### 3.3. UPLC-Q-Exactive-MS Analysis

The identifications were performed on an UPLC-Q-Exactive-MS equipped with an electrospray ion source (HESI) and Xcalibur 3.0 software (Thermo-Fisher, Waltham, MA, USA). The resolution of the full MS was 70,000 and the resolution of the dd-MS^2^ was 17,500. The full-scan mode (*m*/*z*, 100–1500) and Full MS/dd-MS^2^ in positive ion mode were implemented to establish the MS analysis. Auxiliary gas flow rate was 10 arb, and the ion spray voltage was set at 3.5 KV. Capillary temperature was set at 300 °C. The collision energy was set to n (CE) 20, 30, and 50 eV.

The chromatographic column was Acquirty UPLC BEH C_18_ (2.1 mm × 50 mm, 1.7 µm). The column temperature was controlled at 30 °C. The mobile phase was acetonitrile (B) and 0.01% formic acid water (A). The flow rate was 0.25 mL/min. The injection volume was 2 μL. The mobile phase elution system was performed in a linear gradient (19–22% B at 0–3 min, 22–30% B at 3–6 min, 30–32% B at 6–9 min, 32–35% B at 9–16 min, 35–55% B at 16–18 min, 55% B at 18–22 min, 55–70% B at 22–26 min, 70–90% B at 26–29 min, 90–19% B at 29–29.1 min, and 19% B at 29.1–32 min)**.**

### 3.4. Simultaneous Determination of Individual Ginsenosides

Analyses were performed on a CXTH-3000 HPLC system. The mobile phase consisted of water (A) and acetonitrile (B) at a flow rate of 1.0 mL/min through an Agilent 5 HC-C_18_ column (250 × 4.6 mm). The mobile phase elution system was performed in a linear gradient (22–27% B at 0–10 min, 27–30% B at 10–15 min, 30–32% B at 15–35 min, 32–35% B at 35–40 min, 35–40% B at 40–43 min, 40–46.9% B at 43–48 min, 46.9–47.6% B at 48–50 min, 47.6–48.5% B at 50–65 min, 48.5–53% B at 65–68 min, 53–65% B at 68–73 min, 65–85% B at 73–78 min, 85% B at 78–83 min, 85–22% B at 83–85 min, and 22% B at 85–95 min). The column temperature was kept at 30 °C, the UV detection wavelength was 203 nm, and the injection amount was 20 μL.

### 3.5. Optimization of Extraction Process

#### 3.5.1. Extraction Method

All samples were ground into powder sieved by an 100-mesh sieve. We weighed 1.0 g ginseng powder and mixed it with different solvents of different volumes. It was placed in ultrasonic extractor and extracted at different time. After extraction, the ginseng powder residue was filtered and discarded, and the filtrate was obtained. Total saponins were calculated.

#### 3.5.2. Selection of Variables

The content of total ginsenosides extracted by ultrasonic extraction was affected by many factors, such as solvent type, solvent concentration, liquid–solid ratio, and extraction time. In order to select the suitable variable range, single factor experiments were carried out on the solvent type (methanol, ethanol, N-butanol), solvent concentration (50–90%), liquid–solid ratio (10–50 mL/g), and extraction time (20–60 min). The total saponins were extracted under different conditions, one factor was changed each time, and the other three factors remained unchanged.

#### 3.5.3. Selection of Various Factors and Experimental Design

The experiment investigated the three influencing factors of ethanol concentration (A), the ratio of liquor to material (B), and extraction time (C). According to the optimal level of each factor selected by the single factor experiment, the three influencing factors in the response surface method experiment and the level settings are shown in Table 5.

#### 3.5.4. Total Ginsenoside Content of Ginseng

To find the total ginsenoside content, one must accurately draw 1 mL of the test solution into a test tube with a stopper glass, and evaporate the solvent in a water bath at 90 °C. After the solvent is completely evaporated, remove it from the water bath. Precisely add 0.2 mL vanillic aldehyde glacial acetic acid solution and 0.8 mL perchloric acid solution, shake well, place in a constant temperature water bath at 60 °C for 15 min, remove and rinse with cold water, and quickly reduce the tube temperature to room temperature; then, add 5 mL glacial acetic acid, shake evenly, and let stand for 30 min at room temperature. Using the reagent as a blank control, the absorbance value was measured using an ultraviolet spectrophotometer at a wavelength of 550 nm.

## 4. Conclusions

Ginseng is one of the most commonly used traditional Chinese medicine tonics. For decades, the research on its quality control methods has never stopped. Modern scientific research has fully proved that ginsenosides almost reflect all the pharmacological effects of ginseng. Due to the different extraction methods of ginseng in the pharmacopoeias of the four countries, CP, EP, and USP all involve the heating process in the process of extracting ginseng samples. Ginseng is extracted by heating, and the activation energy provided by the solution changes the chemical structure of ginsenoside. The chemical structure diversity of rare ginsenosides is richer, which reflects the diversity of biological activities of ginseng. However, ginsenoside is transformed after heating, which affects the accuracy of the experimental results. The quantitative method of ginseng is insufficient, and the quality identification of ginseng has defects, which cannot reflect the real content of ginsenoside. This paper compares and analyzes the similarities and differences of ginseng content detection methods in CP, USP, EP, and J/KP. The effects of four pharmacopoeia pretreatment methods on ginsenoside content and the optimization of ginsenoside extraction process were investigated. It was illustrated that the pretreatment method has a significant influence on the content determination of ginseng. The yield of rare saponins was increased by heating concluded from both the qualitative and quantitative comparison, and a simple and feasible extraction method was optimized by response surface method at room temperature. It provides important references for the revision of ginseng quality standards.

## Figures and Tables

**Table 1:** Content of ginsenoside in different pharmacopoeias.

	CP	USP	EP	J/KP
Rg_1_ (mg/g)	0.43 ± 0.022	0.46 ± 0.026	0.49 ± 0.029	0.70 ± 0.037
Re (mg/g)	1.80 ± 0.091	1.87 ± 0.099	1.96 ± 0.112	2.25 ± 0.122
Rh_1_ (mg/g)	0.21 ± 0.021	0.20 ± 0.015	0.18 ± 0.011	0.04 ± 0.002
Rb_1_ (mg/g)	2.01 ± 0.102	2.26 ± 0.106	2.31 ± 0.112	2.42 ± 0.116
Rc (mg/g)	0.23 ± 0.013	0.20 ± 0.011	0.20 ± 0.012	0.11 ± 0.006
F1 (mg/g)	0.05 ± 0.004	0.05 ± 0.003	0.04 ± 0.002	0.01 ± 0.001
Rd (mg/g)	0.39 ± 0.023	0.30 ± 0.022	0.22 ± 0.015	0.06 ± 0.003
Rg_3_ (mg/g)	0.02 ± 0.001	0.01 ± 0.001	0.01 ± 0.001	-
Rg_5_ (mg/g)	0.09 ± 0.006	0.04 ± 0.002	0.02 ± 0.001	-
Rh_2_ (mg/g)	0.01 ± 0.001	0.01 ± 0001	0.01 ± 0.001	-
PPT (mg/g)	-	-	-	-
PPD (mg/g)	-	-	-	-

**Table 2:** The BBD matrix and the obtained results at different levels of the experimental variables.

No.	A: Ethanol Concentration (%)	B: The Ratio of Liquid to Material (mL/g)	C: Extraction Time (min)	Y: Total Saponin Content (mg/g)
1	80	20	40	34.25 ± 0.86
2	70	30	40	35.12 ± 0.97
3	70	20	30	42.33 ± 1.05
4	70	20	30	41.65 ± 1.16
5	60	20	40	28.50 ± 0.59
6	70	10	20	34.00 ± 0.82
7	80	30	30	34.16 ± 0.75
8	60	30	30	29.88 ± 0.68
9	80	10	30	33.48 ± 1.03
10	70	30	20	35.82 ± 0.68
11	70	10	40	36.89 ± 0.96
12	60	20	20	32.25 ± 0.72
13	60	10	30	30.59 ± 0.78
14	70	20	30	41.23 ± 0.99
15	70	20	30	42.12 ± 1.21
16	70	20	30	42.27 ± 1.12
17	80	20	20	33.67 ± 0.72

**Table 3:** Analysis of variance of experimental regression equation.

Source	Sum of Squares	DF	Mean Square	F Value	*p-*Value Prob > F	Significant
Model	331.17	9	36.80	57.94	<0.0001	**
A	25.59	1	25.59	40.30	0.0004	**
B	4.51 × 10^−5^	1	4.51 × 10^−5^	7.11 × 10^−5^	0.9935	
C	0.12	1	0.12	0.18	0.6830	
AB	0.49	1	0.49	0.77	0.4089	
AC	4.63	1	4.63	7.29	0.0307	*
BC	3.21	1	3.21	5.06	0.0593	
A^2^	182.79	1	182.79	287.80	<0.0001	**
B^2^	45.89	1	45.89	72.25	<0.0001	**
C^2^	41.94	1	41.94	66.03	<0.0001	**
Residual	4.45	7	0.64			
Lack of Fit	3.56	3	1.19	5.34	0.0697	
Pare Error	0.89	4	0.22			
Cor Total	335.62	16				
Std. Dev	0.80	R^2^	0.9868			
Mean	35.78	Adj R^2^	0.9697			
C.V. %	2.23	Pred R^2^	0.8263			
PRESS	58.31	Adeq precision	20.825			

* Significant at *p* < 0.05, ** Significant at *p* < 0.01.

**Table 4:** The main difference of the pre-processing in the four pharmacopoeias.

Pharmacopoeia Name	Extraction Method	Temperature	Heating Time	Reagent
CP	Trichloromethane soxhlet and butannol ultrasonic extraction	60 °C	3 h	Trichloromethane; Butanol
USP	Water bath reflux extraction	100 °C	1 h	Ethanol-water (4:6)
EP	Water bath reflux extraction	100 °C	1 h	Methanol-water (50:50); Acetonitrile-water (20:80)
J/KP	Shake extraction	Room temperature	-	Methanol-water (3:5); Dilute sodium hydroxide solution; 0.1 mol/L Hydrochloric acid solution

**Table 5:** Experimental variables and their levels in Box–Behnken design.

Factor	Level		
	Low (−1)	Central (0)	High (1)
A: Ethanol concentration (%)	60	70	80
B: The ratio of liquor to material (mL/g)	10	20	30
C: Extraction time (min)	20	30	40

## Supplementary Materials

The following supporting information can be downloaded at: https://www.mdpi.com/1420-3049/27/14/4347/s1, Figure S1: Quantitative chromatogram of ginseng using the four-country pharmacopoeia extraction method; Figure S2: Chemical structures and possible transformation of ginsenosides in heated ginseng; Figure S3: The structure of ginsenosides identified by UPLC-Q-Exactive-MS; Figure S4: 2D contour plots showing the effects of ethanol concentration, liquid to material ratio, and extraction time on the extraction yield of ginsenoside content and their mutual effects; Table S1: UPLC-Q-Exactive-MS analysis.
