# Supplementary material for: Effects of Different Extraction Methods in Pharmacopoeia on the Content and Structure Transformation of Ginsenosides
Source: Molecules. 2022 Jul 6;27(14):4347. doi: 10.3390/molecules27144347 (PMC9351678; doi:10.3390/molecules27144347)
Supplement: Supplementary file 1 [file molecules-27-04347-s001.zip › molecules-1790426-supplementary.pdf]

Supplementary data

Effects of Different Extraction Methods in Pharmacopoeia on the Content and Structure Transformation of Ginsenosides

Hui Li [1], Hua Jiang [1], Lei Xu [1], Yaling Deng [1], Jing Xu [1,*] and Yuqing Zhao [1,2,*]

[1] School of Functional Food and Wine, Shenyang Pharmaceutical University, Shenyang 110016, China; 18844737860@163.com (H.L.); hahajiang6388@163.com (H.J.); 18246691730@163.com (L.X.); yl18341473234@163.com (Y.D.)

[2] Key Laboratory of Natural Medicines of the Changbai Mountain, Ministry of Education, Yanbian University, Yanji 133002, China

* Correspondence: xj19873251@163.com (J.X.); 107000102@syphu.edu.cn (Y.Z.); Tel.: +86-24-43520309 (Y.Z.); Fax: +86-24-435230300 (Y.Z.)

Figure S1. Quantitative chromatogram of ginseng using the four-country pharmacopoeia extraction method.

Figure S2. Chemical structures and possible transformation of ginsenosides in heated ginseng.

Figure S3. The structure of ginsenosides identified by UPLC-Q-Exactive-MS.

Figure S4. 2D contour plots showing the effects of ethanol concentration, liquid to material ratio, and extraction time on the extraction yield of ginsenoside content and their mutual effects.

Table S1. UPLC-Q-Exactive-MS analysis

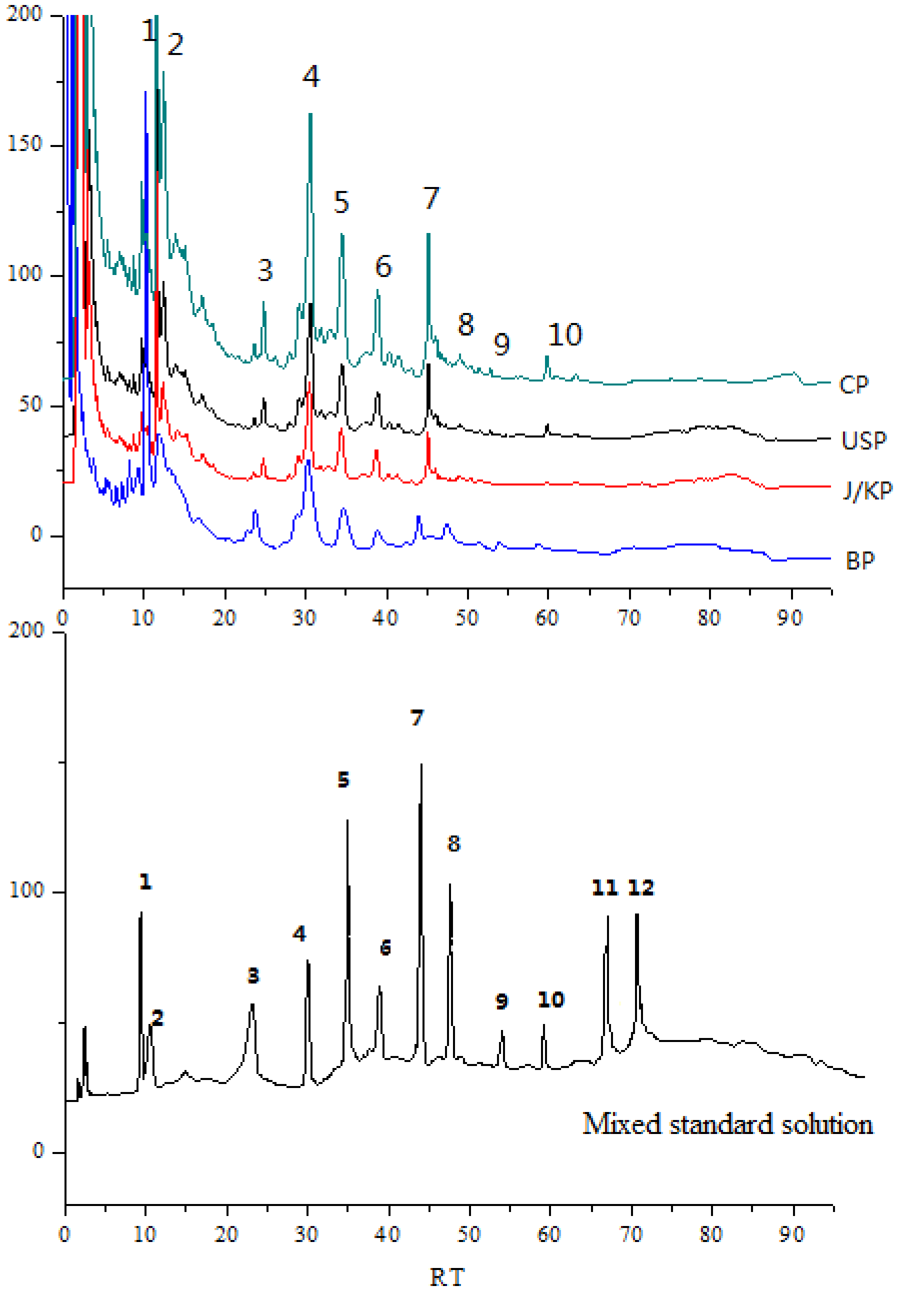

1. Rg_1 2. Re 3. Rh_1 4. Rb_1 5. Rc 6. F1 7. Rd 8. Rg_3 9. Rg_5 10. Rh_2 11. PPT 12. PPD

Fig. S1. Quantitative chromatogram of ginseng using the four-country pharmacopoeia extraction method

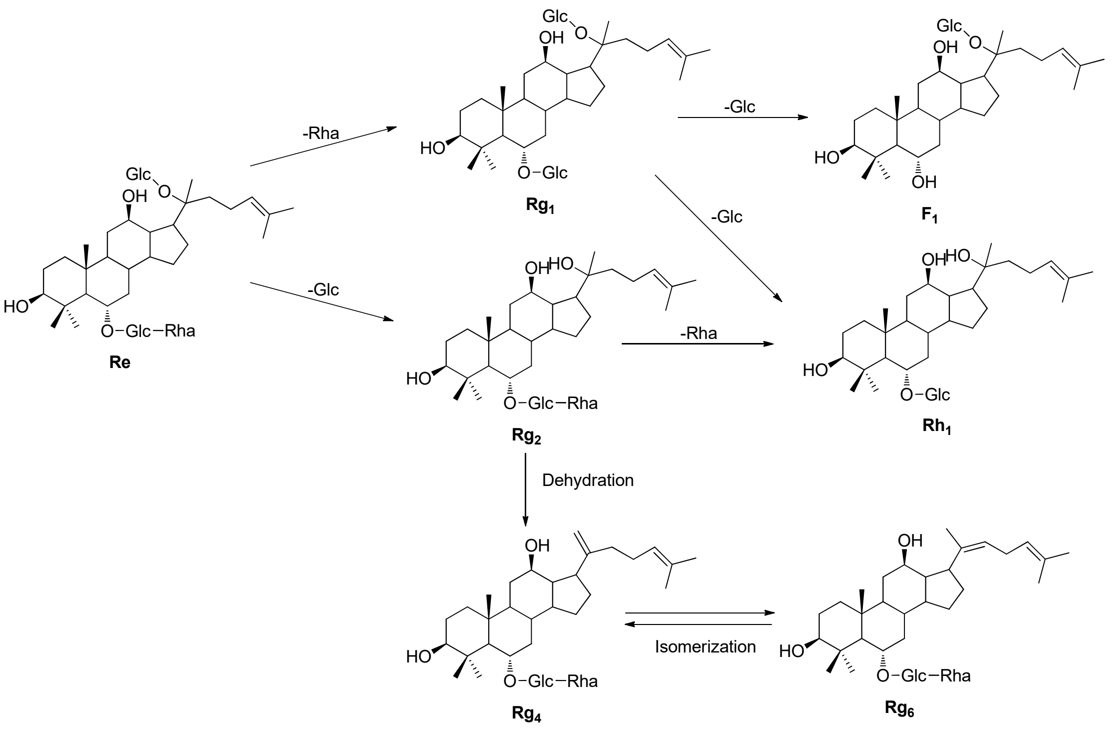

Fig. S2. Chemical structures and possible transformation of ginsenosides in heated ginseng.

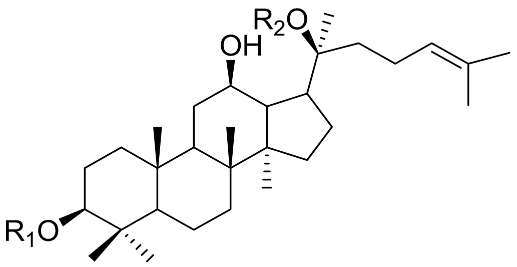

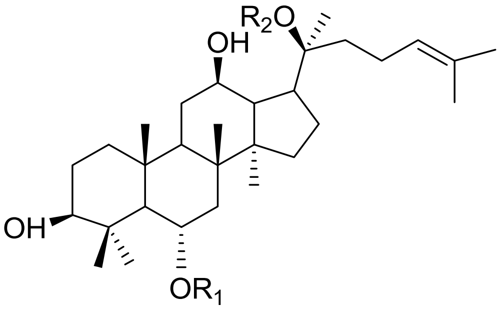

PPD-type

PPT-type

Name	R_1	R_2
Ra_1	Glc2-Glc	Glc6-Ara（p）4-Xyl
Ra_3	Glc2-Glc	Glc6-Glc3-Xyl
Rb_1	Glc2-Glc	Glc6-Glc
Rb_2	Glc2-Glc	Glc6-Ara（p）
Rb_3	Glc2-Glc	Glc6-Xyl
Rc	Glc2-Glc	Glc6-Ara（f）
Rd	Glc2-Glc	Glc
Noto-Fe	Glc	Glc6-Ara（f）
F_2	Glc	Glc
20（S）-Rg_3	Glc2-Glc	H
20（R）-Rg_3	Glc2-Glc	H
Noto-R_3	Glc2-Xyl	Glc
PPD	H	H

Name	R_1	R_2
Re	Glc2-Xyl	Glc
Rg_1	Glc	Glc
Rg_2	Glc2-Rha	H
F_1	H	Glc
F_5	H	Glc2-Glc
F_3	H	Glc6-Ara（p）
Mc	H	Glc6-Ara（f）
Rf	Glc2-Glc	H
Rh_1	Glc	H
PPT	H	H

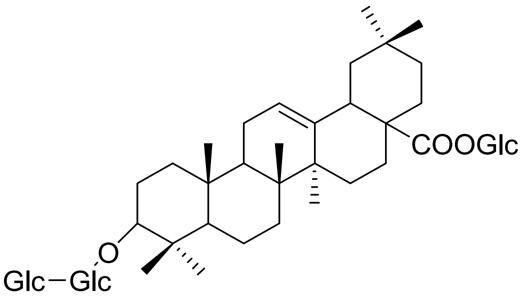

Ginsenoside Ro

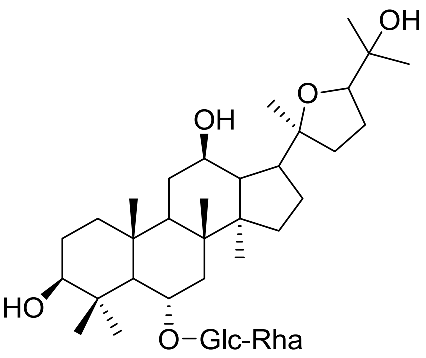

pseudo-ginsenoside F_{11}

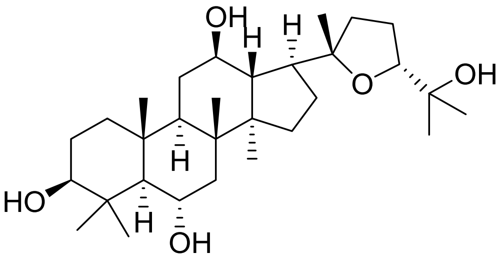

Pseudo sapogenin

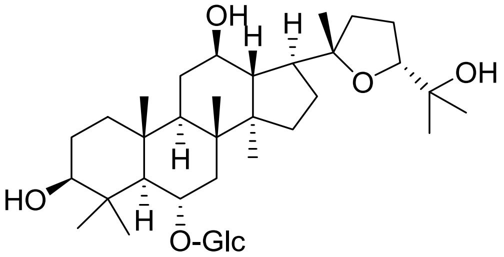

RT_5

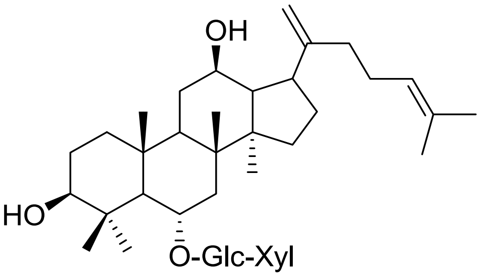

Noto-T5

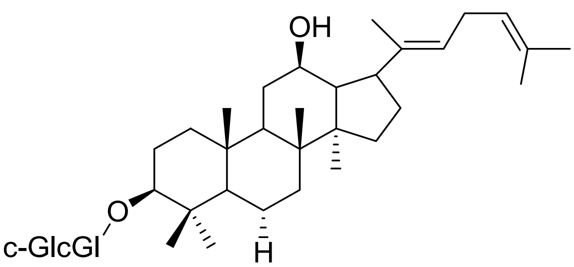

Rg_5

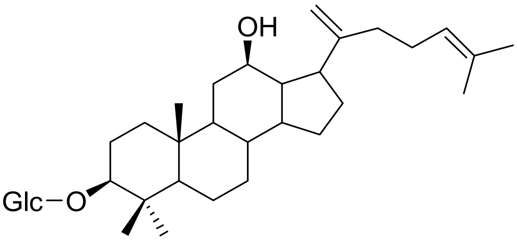

Rk_2

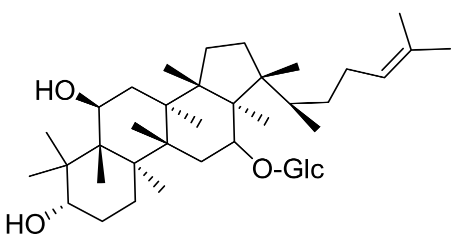

Rh_8

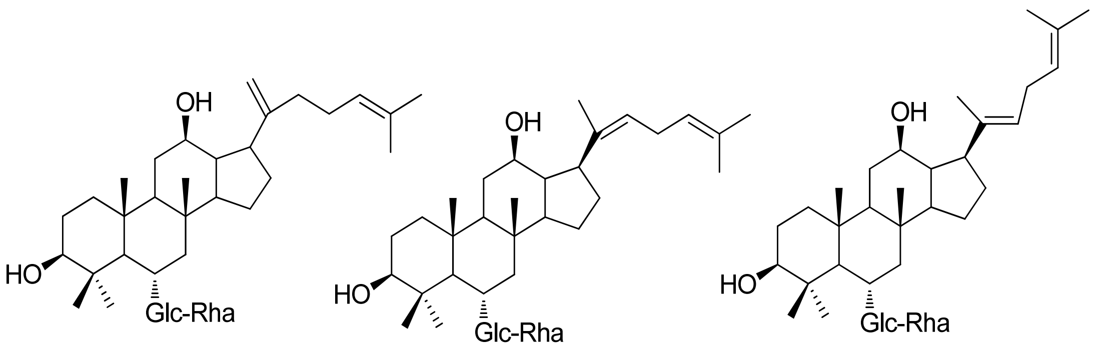

Rg$_4$ **Rg$_6$** **F$_4$**

Fig. S3. The structure of ginsenosides identified by UPLC-Q-Exactive-MS

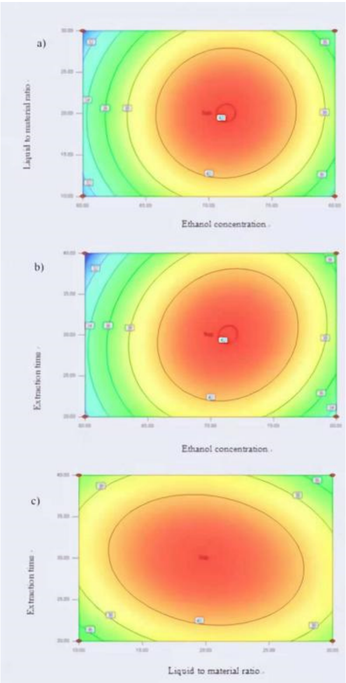

Fig. S4. 2D contour plots showing the effects of ethanol concentration, liquid to material ratio, and extraction time on the extraction yield of ginsenoside content and their mutual effects.

Table S1. UPLC-Q-Exactive-MS analysis

Peak	Identity	t_R/min	Molecular formula	Extracting Ion(m/z)[M+H]$^+$
1	Ginsenoside Ra_3	11.635	$C_{59}H_{100}O_{27}$	1241.6511
2	Ginsenoside Ra_1	12.786	$C_{58}H_{98}O_{26}$	1211.6404
3	Ginsenoside Rb_1	11.581	$C_{54}H_{92}O_{23}$	1109.6086
4	Ginsenoside Rc	12.582	$C_{53}H_{90}O_{22}$	1079.5985
5	Ginsenoside Rb_2	13.877	$C_{53}H_{90}O_{22}$	1079.5985
6	Ginsenoside Rb_3	18.413	$C_{53}H_{90}O_{22}$	1079.5985
7	Notoginsenoside R_3	10.118	$C_{48}H_{82}O_{19}$	963.55109
8	Ginsenoside Ro	13.026	$C_{48}H_{76}O_{19}$	957.50439
9	Ginsenoside Re	11.661	$C_{48}H_{82}O_{18}$	947.5571

10	Ginsenoside Rd	18.538	$C_{48}H_{82}O_{18}$	947.5571
11	Notoginsenoside Fe	13.961	$C_{47}H_{80}O_{17}$	917.5463
12	Quinquefolium Saponin L_{10}	19.062	$C_{47}H_{80}O_{17}$	917.5463
13	Ginsenoside Rg_1	5.228	$C_{42}H_{72}O_{14}$	801.4981
14	Ginsenoside Rf	6.593	$C_{42}H_{72}O_{14}$	801.4981
15	pseudo-ginsenoside F_{11}	10.12	$C_{42}H_{72}O_{14}$	801.4981
16	Ginsenoside Rg_2	13.819	$C_{42}H_{72}O_{13}$	785.5009
17	Ginsenoside F_2	16.824	$C_{42}H_{72}O_{13}$	785.5009
18	(S)-Ginsenoside Rg_3	11.613	$C_{42}H_{72}O_{13}$	785.5009
19	(R)-Ginsenoside Rg_3	12.198	$C_{42}H_{72}O_{13}$	785.5009
20	Ginsenoside F_3	11.302	$C_{41}H_{70}O_{13}$	793.4688 $[M+Na]^+$
21	Ginsenoside F_5	11.302	$C_{41}H_{70}O_{13}$	793.4688 $[M+Na]^+$
22	Ginsenoside Rg_5	7.207	$C_{42}H_{70}O_{12}$	767.4339
23	Ginsenoside Rg_4	9.679	$C_{42}H_{70}O_{12}$	767.4339
24	Ginsenoside Rg_6	14.779	$C_{42}H_{70}O_{12}$	767.4339
25	Ginsenoside F4	18.056	$C_{42}H_{70}O_{12}$	767.4339
26	Ginsenoside MC	13.921	$C_{41}H_{70}O_{12}$	755.4929
27	Notoginsenoside T_5	10.849	$C_{41}H_{68}O_{12}$	753.4447
28	RT_4	1.004	$C_{36}H_{62}O_{10}$	655.44043
29	RT_5	10.169	$C_{36}H_{62}O_{10}$	655.44043
30	Ginsenoside F_1	1.554	$C_{36}H_{62}O_9$	639.4464

31	Ginsenoside Rh_1	17.095	$C_{36}H_{62}O_9$	639.4464
32	Rh_8	1.071	$C_{36}H_{60}O_9$	637.1878
33	Ginsenoside RK_2	12.783	$C_{36}H_{60}O_7$	605.4405
34	pseudo-sapogenin	19.341	$C_{30}H_{52}O_5$	515.3712 $[M+Na]^+$
35	PPT	28.991	$C_{30}H_{52}O_4$	477.3933
36	PPD	23.813	$C_{30}H_{52}O_3$	461.3989
